# Intense natural selection preceded the invasion of new adaptive zones during the radiation of New World leaf-nosed bats

**DOI:** 10.1038/s41598-017-08989-6

**Published:** 2017-09-11

**Authors:** Daniela M. Rossoni, Ana Paula A. Assis, Norberto P. Giannini, Gabriel Marroig

**Affiliations:** 1 Department of Genetics and Evolutionary Biology, Biosciences Institute, University of São Paulo, Rua do Matão, 277, 05508-900 São Paulo, Brazil; 2 Department of Ecology, Biosciences Institute, University of São Paulo, Rua do Matão, 277, 05508-900 São Paulo, Brazil; 3 Unidad Ejecutora Lillo-CONICET, Miguel Lillo 251, Universidad Nacional de Tucumán, Tucumán, 4000 Argentina

## Abstract

The family Phyllostomidae, which evolved in the New World during the last 30 million years, represents one of the largest and most morphologically diverse mammal families. Due to its uniquely diverse functional morphology, the phyllostomid skull is presumed to have evolved under strong directional selection; however, quantitative estimation of the strength of selection in this extraordinary lineage has not been reported. Here, we used comparative quantitative genetics approaches to elucidate the processes that drove cranial evolution in phyllostomids. We also quantified the strength of selection and explored its association with dietary transitions and specialization along the phyllostomid phylogeny. Our results suggest that natural selection was the evolutionary process responsible for cranial diversification in phyllostomid bats. Remarkably, the strongest selection in the phyllostomid phylogeny was associated with dietary specialization and the origination of novel feeding habits, suggesting that the adaptive diversification of phyllostomid bats was triggered by ecological opportunities. These findings are consistent with Simpson’s quantum evolutionary model of transitions between adaptive zones. The multivariate analyses used in this study provides a powerful tool for understanding the role of evolutionary processes in shaping phenotypic diversity in any group on both micro- and macroevolutionary scales.

## Introduction

A fundamental challenge in evolutionary biology is to understand the processes underlying the astonishing phenotypic diversity seen today. Adaptive radiations provide valuable material for the study of evolutionary processes because they rapidly produce a wealth of adaptations to different environments and increase species diversity^1–4^. An adaptive radiation event occurs when natural selection and ecological opportunity interact, favouring the rapid appearance of new lineages with distinct adaptations that enable exploitation of different resources or niches, resulting in increased taxonomic, ecological, and phenotypic diversity^1, 2, 5–7^.

The New World leaf-nosed bats, family Phyllostomidae, are arguably the most ecologically diverse group of mammals^6^. Phyllostomids have a distribution ranging from southern Arizona and the West Indies to northern Argentina, and have been highly successful in exploiting a diverse array of resources^6^. These bats have evolved over the last 30 million years^8^, and their dietary breadth and feeding habits are exceptionally diverse compared with other mammals; the family includes species highly specialized for feeding on insects, vertebrates, fruit, pollen, nectar, young leaves, and even blood^6^. This ecological diversification is reflected in phyllostomid morphology, which shows wide variation in body size and shape, particularly in the skull. The phyllostomid skull provides an excellent model with which to test hypotheses on the evolution of complex morphological phenotypes because of the various functions it performs and its dynamic developmental complexity. Due to its impressive morphological diversity, its ecological importance, and its relevance for the innovation of echolocation by nasal emission, the phyllostomid skull has been extensively studied at a functional, biomechanical, and developmental level^9–27^. The remarkable specializations seen in these bats provide a unique opportunity for investigation of the evolutionary forces that have promoted such striking phenotypic diversity and the intensity of these forces.

Here, we used comparative quantitative genetics approaches to investigate the evolutionary processes underlying the diversification of cranial morphology in New World leaf-nosed bats. Specifically, we tested whether the cranial diversity seen in phyllostomids can be explained solely by genetic drift, or whether natural selection played an important role in its evolution. Seeking to integrate quantitative genetics with the ecological theory of adaptive radiation, we also addressed the following questions: firstly, if phyllostomid diversification was adaptive, then how strong was selection during morphological evolution? and secondly, are the greatest magnitudes of selection associated with the invasion of novel ecological niches? We hypothesised that the phyllostomid radiation might have been favoured by directional selection for successful exploitation of new ecological opportunities. Therefore, we expected the evolution of dietary specializations and novel feeding habits along the phyllostomid phylogeny to be associated with stronger selection magnitudes.

## Results

### Evolutionary processes responsible for the diversification of phyllostomid bats

In order to assess the evolutionary processes responsible for the diversification of cranial morphology in phyllostomid bats, we used two drift tests, a regression test and a correlation test (see the Methods section for a detailed description of these neutrality tests). Both tests are grounded in quantitative genetics theory and are based on the expectation that under neutral evolution, the direction and magnitude of multivariate variance-covariance evolution should be proportional to the ancestral covariance patterns^28–30^.

The neutrality tests showed that the cranial diversity of phyllostomid bats is unlikely to have been produced by genetic drift alone (Tables  1, 2 and Supplementary Fig. S1). The regression tests revealed nine nodes with a regression slope significantly different from one, suggesting that non-random forces like natural selection played an important role in the observed morphological patterns, particularly in the deepest nodes (Table 1 and Supplementary Fig. S1). The regressions of between-groups variances (**B**) on within-group eigenvalues (**W**) and the associated 95% confidence intervals for nodes where genetic drift was rejected as the sole explanation of phenotypic variation are presented in Supplementary Fig. S2. These plots show that significant deviations in regression slopes from expectations based on drift were largely due to variation in the first principal component (PC1).

**Table 1:** Results for regression drift test. Boldface numbers indicates regression coefficients significantly different from 1.0. The label (b), represents the slope of the regression line estimated between **W**-matrix and B-matrix for the regression test with the respective confidence intervals (b 95% intervals). Node labels match those of *SI Appendix*, Fig. S1.

**Taxon/Node labels**	**N taxa**	**N skulls**	**b**	**b 95% interval**
Phyllostomidae (58)	**57**	**2808**	**1.216**	**1.042<>1.391**
59	**55**	**2689**	**1.218**	**1.040<>1.397**
60	**53**	**2615**	**1.222**	**1.042<>1.402**
63	**50**	**2414**	**1.207**	**1.030<>1.384**
64	**41**	**1947**	**1.181**	**1.011<>1.351**
Glossophaginae + Lonchorhininae (65)	**13**	**1868**	**1.331**	**1.195<>1.466**
Glossophaginae (66)	**11**	**564**	**1.317**	**1.175<>1.458**
67	5	159	1.036	0.881<>1.190
71	6	326	1.115	0.958<>1.273
77	28	1383	1.137	0.958<>1.315
80	25	1249	1.113	0.923<>1.303
Rhinophyllinae + Stenodermatinae (83)	22	1115	1.120	0.919<>1.320
Stenodermatinae (84)	21	1063	1.124	0.931<>1.317
85	20	1030	1.132	0.944<>1.320
86	**13**	**619**	**1.202**	**1.029<>1.375**
87	12	557	1.176	0.976<>1.376
88	11	546	1.164	0.965<>1.362
Subtribe Stenodermatina (89)	8	410	0.901	0.715<>1.088
98	7	411	1.092	0.918<>1.266
99	6	351	1.038	0.838<>1.237
Phyllostominae (104)	**9**	**467**	**1.238**	**1.093<>1.382**
106	7	368	1.185	0.977<>1.392
107	5	257	1.116	0.902<>1.329

**Table 2:** Results for Principal Components (PC) correlation drift test. The first number indicates a specific PC and the subsequent numbers in parentheses are the PCs to which a significant correlation was found (p < 0.01). Node labels match those of *SI Appendix*, Fig. S1.

**Taxon/Node labels**	**Correlated PCs**	**Drift Rejected**
Phyllostomidae (58)	1(2;3;5;6;9;10); 2(3;5;6;9;10); 3(4;5;8;9;10); 4(5;8;9;10);	**yes**
	5(9;10); 6(9); 8(9;10); 9(10)	
59	1(2;3;5;6;9;10); 2(3;5;6;9;10); 3(4;5;8;9;10); 4(5;8;9;10);	**yes**
	5(8;9;10); 6(9); 8(9;10); 9(10)	
60	1(2;3;5;6;9;10); 2(3;5;6;9;10); 3(4;5;8;9;10); 4(5;8;9;10);	**yes**
	5(9;10); 6(9); 9(10)	
63	1(2;3;5;6;9;10); 2(3;5;6;9;10); 3(4;5;9;10); 4(5;9;10);	**yes**
	5(9;10); 6(9); 9(10)	
64	1(2;4;5;6;7;8;9;10); 2(4;5;10); 3(4;5;7;9;10); 4(5;7;9;10);	**yes**
	5(7;9;10); 6(8); 7(9;10); 8(9;10); 9(10)	
65	1(2;3;4;5;6;9;10); 2(3;4;5;6;9;10); 3(5;6); 4(5;6;9;10);	**yes**
	5(6;9;10); 7(9); 9(10)	
Glossophaginae (66)	1(2;3;4;5;6;9); 2(3;4;5;6;9); 4(5;6;9); 5(6;9);6(7)	**yes**
67	1(2)	**yes**
71	1(2)	**yes**
77	1(2;3;5;9;10); 3(4;7;9;10); 6(7;8); 7(8); 9(10)	**yes**
80	1(2;3;5;9;10); 3(7;10); 4(9); 6(7;9); 7(8); 9(10)	**yes**
83	1(2;3;5;9); 3(10); 6(7); 8(9)	**yes**
Stenodermatinae (84)	1(2;3;8;9); 3(9); 4(10); 6(7); 8(9)	**yes**
85	1(2;3;8;9); 3(9); 4(10); 6(10)	**yes**
86	1(2;3); 3(10); 4(9); 7(8)	**yes**
87	1(2;6)	**yes**
88	1(2;6)	**yes**
Subtribe Stenodermatina (89)	1(2)	**yes**
98	1(2;4)	**yes**
99		no
Phyllostominae (104)	1(2;3;4;5); 2(3;4;5); 3(4;5)	**yes**
106	1(3;4)	**yes**
107	1(3)	**yes**

The correlation test also rejected the null hypothesis that the observed morphological patterns were caused by genetic drift alone in 22 nodes, including the nine nodes in which the same hypothesis was rejected by the regression test (Table 2 and Supplementary Fig. S1). The null hypothesis of genetic drift was not rejected in only one node (node 99). It is noteworthy that this node encompasses only six species; therefore, the inability to reject drift in this case might be due to lack of statistical power. In each case where the correlation test rejected the null hypothesis of neutral evolution, the first PC appeared to be significantly associated with one or more of the first ten PCs (Table 2). In fact, most of the divergence observed between phyllostomid species is associated with the first principal component of **W** (Supplementary Fig. S3). The eigenvectors estimated for the first ten PCs from the ancestral **W**-matrix (node 58 in the Phyllostomidae phylogeny presented in Supplementary Fig. S1) are provided in Supplementary Table S1. Together, these PCs account for 99.4% of the total variation. PC1 is primarily an allometric size factor, describing variation in cranial size and associated shape, with all of its coefficients consisting entirely of positive or negative values, suggesting that cranial traits all increase or decrease together along its direction (Supplementary Table S1). Additionally, we found a positive and significant correlation between the geometric mean of skull for each species and projection of the species mean onto PC1 of **W**, which confirms that PC1 is a good metric of size (r^2^ = 0.97, Supplementary Table S2 and Supplementary Fig. S4).

### Magnitude of selection and the invasion of new adaptive zones

**Figure 1:**
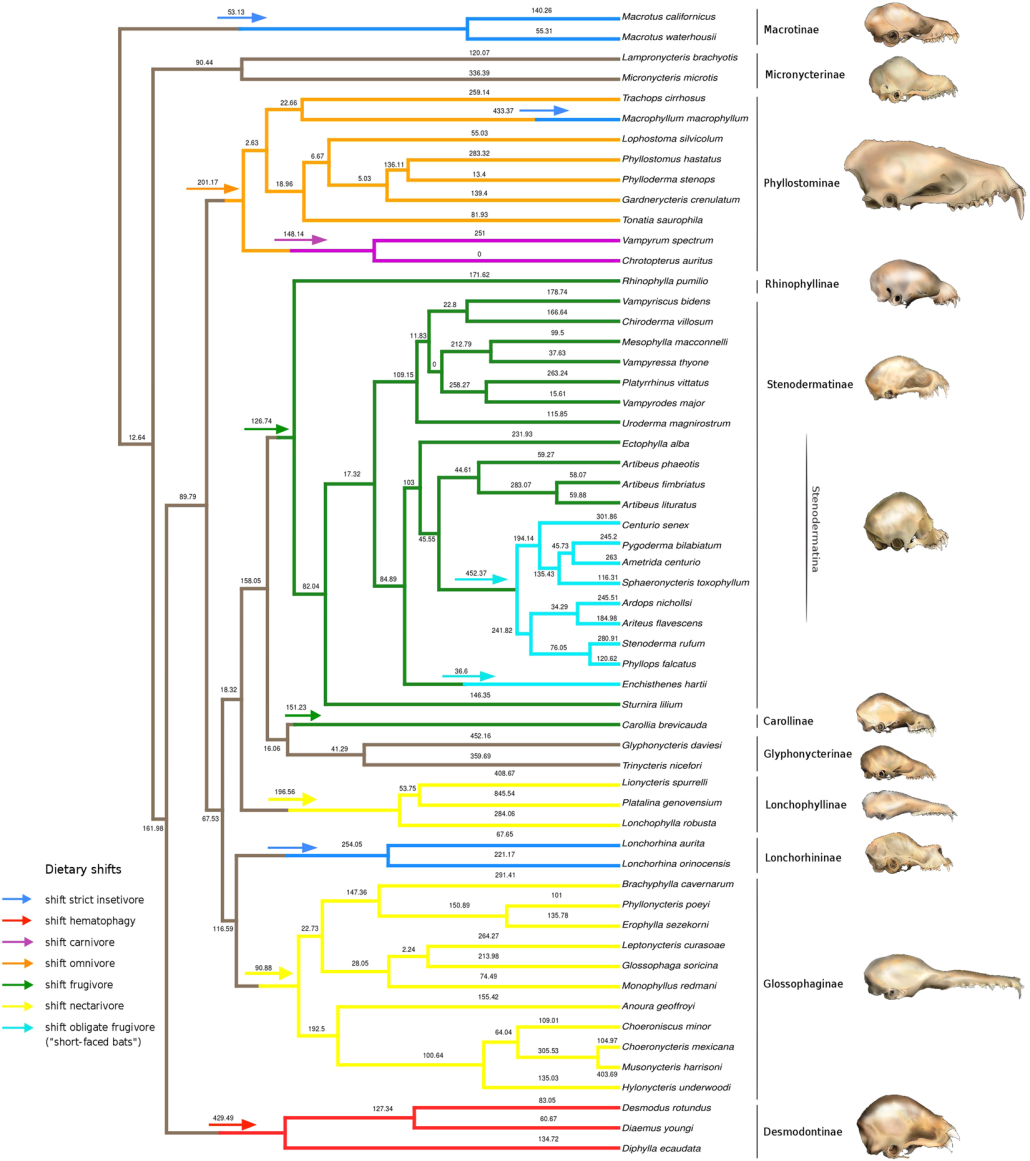
Magnitude of selection, estimated as the norm of the selection gradient vector, along the phylogeny proposed by Rojas^8^. The arrows indicate dietary transitions.

**Figure 2:**
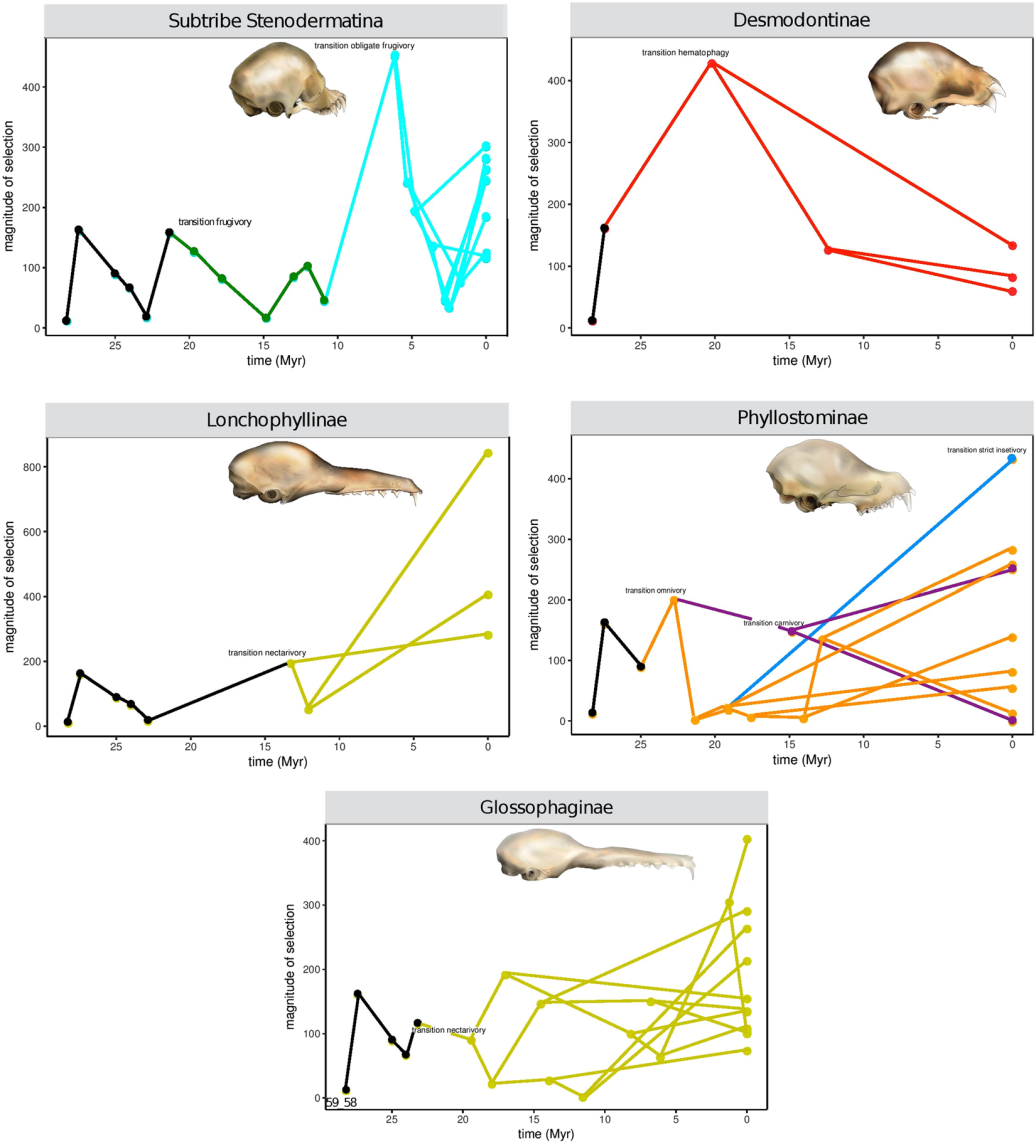
Magnitude of selection, estimated as the norm of the selection gradient vector, plotted against time (Myr) in five representative clades of the Family Phyllostomidae. Skulls: *Sphaeronycteris toxophyllum* (obligate frugivore, Subtribe Stenodermatina), *Desmodus rotundus* (hematophagous, Subfamily Desmodontinae), *Platalina genovensium* (nectarivore, Subfamily Lonchophyllinae), *Trachops cirrhosus* (omnivore, Subfamily Phyllostominae) and *Musonycteris harrisoni* (nectarivore, Subfamily Glossophaginae).

The magnitude of the reconstructed selection gradients, estimated as the norm of the reconstructed selection vector, varied greatly among branches in the phylogeny (Fig. 1). In general, we found that the most ancestral branches exhibited the lowest magnitude of selection estimates, indicating that selection was stronger on the terminal branches (Fig. 1). During the evolution of phyllostomids, the greatest magnitude of selection appeared to be associated with transitions to feeding specializations (Figs 1 and 2). Overall, during transitions to novel feeding habits, the magnitude of selection reaches its highest values and then decays once the new adaptive zone is filled (Fig. 2).

## Discussion

### Evolutionary processes responsible for phyllostomid diversification

Our results rejected genetic drift as a likely evolutionary mechanism for cranial diversification in phyllostomid bats. Moreover, it is interesting to note that most of the significant results of the genetic drift tests encompassed correlations with PC1. The significant correlations between the allometric size factor (PC1) and several other PCs obtained in the correlation test suggest that these independent morphological dimensions were co-selected^31^ during the evolutionary diversification of the family Phyllostomidae. Different studies have reported that functional demands imposed by dietary specializations influenced cranial evolution in the radiation of phyllostomid bats^6, 10–13, 17–20, 32, 33^. Here, the co-selection observed between allometric cranial size (represented by PC1) and the other PCs may reflect a coordinated interaction of these factors in performing some function. For example, the correlation of PC1 and PC2 in the node representing the origin of the short-faced bats (node 89, Table 2 and Supplementary Fig. 1) suggests that allometric size along with a high frontal bone, basicranium, and cranial vault, associated with a short nasal bone and a short and wide palate, were co-selected during the diversification of this clade. The combination of these co-selected traits, associated with the relative size (length and width) of palate *versus* neurocranium, might have been mechanically advantageous and provided concomitantly high bite forces to address the physical challenges imposed by very hard fruits^6, 10–12, 17^.

Our results indicate that most of the cranial diversification that cannot be explained by neutral evolution can be explained by selection on allometric size variation. Size variation has a significant effect on a number of fundamental traits in bats, including foraging behavior^34^ and physiological features, such as basal metabolic rates^35^, flight performance^36^, echolocation parameters^37^, and most life-history strategies^38^. The divergence observed along the first principal component suggests two hypotheses: firstly, that size evolution is a direct consequence of selection on size, or in other words, that the high divergence observed in the morphospace of PC1 is a result of selection in the direction of PC1; and secondly, that patterns of phenotypic covariation (and therefore, the genetic variation that determines them) dominated and biased cranial diversification in phyllostomid bats, redirecting the evolutionary response^39^. These hypotheses will be the subject of a future contribution.

Previous studies using comparative methods based on Ornstein-Uhlenbeck models have suggested that measures of biomechanical performance in phyllostomid bats^10^, and phyllostomid mandibles in particular^19^, have been subject to directional selection. Our results extended these findings through a comparative quantitative genetic investigation of the diversification of the phyllostomid skull, the most taxonomically diverse analysis to date, which showed that directional natural selection was the key mechanism underlying phyllostomid diversification, and provided a quantitative measure of the strength of selection.

### Magnitude of selection and the invasion of new adaptive zones

During phyllostomid evolution the greatest magnitudes of selection were accentuated in transitions to novel feeding specializations (Figs 1 and 2). These results support Simpson’s theory of adaptive zones on an adaptive landscape^5^, and suggest a strong link between ecological opportunities and episodes of strong selection between distinct adaptive peaks. Under this scenario, during the phyllostomid radiation, transitions to feeding specializations corresponded to peaks in the adaptive landscape. Here, strong natural selection allowed bats to use previously unexploited resources in the new adaptive zones. In the phyllostomid phylogeny, transitions to adaptive zones correspond to the origins of major new clades; the similar morphological traits and patterns of habitat use of the member species of each clade are then explained by common ancestry at these nodes (Figs 1 and 2).

Remarkably, one of the strongest magnitudes of selection was observed in the transition to a feeding strategy of obligate frugivory, on the branch leading to the clade of short-faced bats (subtribe Stenodermatina). This clade has undergone further specialization from a primarily frugivorous ancestor, and its radiation to exploit the obligate frugivory niche represents the most recent burst of diversification in the family Phyllostomidae. Previous studies reported a very high mechanical advantage in the extremely short and wide skull of short-faced bats, associated with a high bite force conforming to their ecological specialization towards hard fruits found in the rainforest canopy, from which the bats extracts the juice^6, 10^. The subfamily Stenodermatinae, including the clade of short-faced bats, exhibits the greatest relative numbers of genera and species in the family Phyllostomidae, with feeding strategies ranging from primary to obligate frugivory^40^ (Fig. 1). The origin of this clade was associated with a significant increase in the rate of species diversification^6, 41^, and the evolution of frugivory is thought to have promoted this successful radiation of the family by opening a new adaptive zone^6^. We found that very strong selective pressure preceded the invasion of this new niche of highly specialized frugivores (Figs 1 and 2). Moreover, our findings explain by means of strong magnitude of natural selection, driven by ecological opportunity, the appearance of an evolutionary novelty, or a key innovation in the clade of short-faced bats, reflecting functional specializations for processing hard canopy fruits.

The second largest magnitude of selection observed during dietary transitions in phyllostomid evolution was found in the branch leading to obligate vampires, the Desmodontinae clade (Figs 1 and 2). This lineage of highly specialized blood-feeding bats contains the common vampire bat (*Desmodus rotundus*), which feeds primarily on mammals, and two species that are specialized towards bird blood^40^. Certainly, the impressive adaptation of Desmodontinae to feeding on bird and mammal blood required considerable physiological, morphological, and ecological changes. Our results suggest that during the evolutionary diversification of this group, strong selection was required to achieve the unique features that made the vampire bats successful obligate sanguivores. Once this phenotype was acquired and the new adaptive zone filled out, the magnitude of selection decayed to a background level comparable with other groups (Figs 1 and 2), indicating that these species had reached (or were close to) an adaptive peak and did not require further bursts of strong directional selection.

Our results also reveal that both clades comprising nectarivorous bats (subfamilies Glossophaginae and Lonchophyllinae) presented the widest distribution of selection strength values (Supplementary Fig. S5). Interestingly, in the subfamily Lonchophyllinae, we found that the branch leading to *Platalina genovensium* exhibited the greatest magnitude of selection among all phyllostomids. This pattern reinforces aspects of Simpson’s hypothesis on the dynamics of adaptive zones, specifically, that adaptive zones are expected to change in a changing environment, and that under risk of extinction, organisms must adapt to these changes over time^5^. Consequently, adaptive zones and subzones may have discontinuities between them, and specialized species play a role in creating them^5^. The long-snouted bat, *Platalina*, is an extremely rare and monotypic genus endemic to western Peru, inhabiting mid- to high-elevation arid regions^42^. This species is characterized by an extremely elongated rostrum and tongue, and has an obligate mutualistic association with the columnar cactus *Weberbauerocereus weberbaueri*, on whose pollen and nectar it depends for survival throughout most of its range^43^. Compared to the other specialized forms within its nectarivorous clade, *Platalina* occupied a narrower zone of specialization. The fact that the strongest magnitude of selection was found on this branch suggests that the evolution of this highly specialized skull may be a good example of how selection takes place in response to ecological opportunity, leading to extreme specialization.

## Conclusion

In this work, we applied multivariate quantitative analysis to investigate the evolutionary mechanisms underlying the cranial diversity observed in New World leaf-nosed bats. The disparity of skull morphology in phyllostomids has long been recognized as a result of adaptive radiation, a concept that depends on the availability of ecological opportunities and the action of directional natural selection. Here, we provided evidence for the hypothesis that natural selection was a major force driving the evolutionary history of this group, directly affecting the morpho-functional structure of the skull. Our study reveals that during the adaptive radiation of phyllostomids, natural selection, triggered by ecological opportunities, favoured the appearance of evolutionary novelties or key morphological innovations in different lineages. In addition, we showed that the changes in selection strength that occurred along the phylogeny were associated with striking transitions in feeding strategies. Furthermore, our results support Simpson’s phenotypic landscape model and demonstrate a strong link between ecological opportunity and a high magnitude of selection, which is accentuated in transitions to novel specialized feeding habits. To our knowledge, this is the first study using the framework of quantitative genetics to reconstruct net selection gradients for such a morphologically diverse clade, with an explicit link to shifts in dietary strategies on a macroevolutionary scale. The multivariate analysis used in this study provides a powerful tool for understanding the roles of adaptive radiations and evolutionary processes in shaping phenotypic diversity in any group of interest, on both micro- and macroevolutionary scales. The integration of concepts from quantitative genetics with knowledge from adjacent disciplines represents a very promising research strategy for understanding the evolution of multidimensional phenotypes in nature and the coexistence of related species in diverse habitats.

## Methods

### Taxon samples

We obtained 35 cranial measurements from 2808 specimens, representing 53 genera and 57 species of phyllostomid bats deposited in the following institutions: Museu de Zoologia da Universidade de São Paulo (MZUSP, São Paulo, SP), Coleção de Chiroptera de São José do Rio Preto (DZSJRP, São José do Rio Preto, SP), Museu Nacional (MN, Rio de Janeiro, RJ), Museu Paraense Emílio Goeldi (MPEG, Belém, PA), Field Museum of Natural History (FMNH, Chicago, IL), National Museum of Natural History (NMNH, Washington, D.C.), American Museum of Natural History (AMNH, New York, NY), Museum of Texas Tech University (TTU, Lubbock, TX) and Museum of Vertebrate Zoology (MVZ, Berkeley, CA). *SI Appendix*, Table S3 provides the sample sizes per species measured in this study. The phyllostomid phylogeny was obtained from Rojas *et al*.^8^. We followed the subfamily taxonomic classification proposed by Baker *et al*.^40^ in which 11 subfamilies, 12 tribes and 56 genera are recognized.

### Data acquisition and covariance matrix estimation

We digitized 3D coordinates for 21 landmarks in each specimen (*SI Appendix*, Fig. S6, Tables S4 and S5) using a Microscribe MX 3D. We then calculated 35 linear distances based on these landmarks and used those to represent the overall cranial morphology. These set of landmarks and the derived inter-landmark reflect important developmental and functional relationships among cranial elements^44–46^ (*SI Appendix*, Table S5). We included only adult specimens in the analysis characterized by relatively complete fusion of the basisphenoid and basioccipital joints (*synchondroses intersphenoidalis and spheno-occipitalis*). When measurements for both sides of the skull were available, bilaterally symmetrical measurements were averaged between sides, and if the skull was damaged on one side, the other side was used instead of the average. Each specimen was digitized twice and repeatability was estimated to assess measurement reliability^47^. All subsequent analyses were carried using the average of replicated measurements.

Pooled within-species phenotypic variance-covariance matrix (**P**-matrices; see *SI Appendix*, Table S6 to check the list of symbols used throughout the text and their respective descriptions) was estimated for each species. In a few cases where sample sizes were too small to confidently estimate a covariance matrix ( < 35 specimens), we used the estimated matrix derived from the immediate ancestral node. Geographic variation, sex, and their possible interaction were evaluated through multivariate analysis of variance (MANOVA), with models chosen on the basis of the Wilk’s lambda statistic, with alpha level of significance set at α = 0.05. In those cases where sources of variation significantly influenced the data, the covariance matrices were estimated using the residual matrix of a general linear model, including the 35 distances as dependent variables and significant sources of variation as independent ones. In cases where no effect was detected, the covariance matrices were estimated directly from the raw data. We calculated the ancestral within-group covariance matrices (**W**-matrices; *SI Appendix*, Table S6) for each node of the phylogeny as an average of the species **P**-matrices, weighted by species sample sizes; using function PhyloW in package “evolqg” for R^48, 49^.

### Overview of the underlying assumptions in a quantitative genetic approach

Our choice to use quantitative genetics as the theoretical framework for this study was because this approach incorporates the genetic covariance between traits in the model. In other words, it takes into account the effect of genetic correlations on the evolutionary outcomes, an important aspect in the present study considering a highly dimensional interdependent multivariate system (35 cranial traits). Moreover, even though Ornstein-Uhlenbeck (OU) comparative methods are also a powerful tool in the investigation of macroevolutionary processes, most of the developed methods are aimed at the investigation of univariate variables, which hinder the application of this methodology to multivariate complex systems as the one we are interested in this study. The methods described below for the study of multivariate phenotypic evolution (the neutrality tests and the net selection gradients reconstructions) are grounded on the assumptions that the patterns of additive genetic variance-covariance matrix (**G**-matrix) from the ancestral to descendant populations have remained relatively stable throughout a clade diversification^28^. However, obtaining accurate estimates of **G** in a multidimensional system is a hard task because it has to be estimated with hundreds, or thousands of specimens with known genealogies^50, 51^. As a consequence, broad scale comparisons of **G** are frequently hampered by practical limitations imposed by experimental designs. In contrast, phenotypic matrices (**P**) are much easier to obtain, as they require relatively smaller sample sizes and no information regarding genealogy^52^. The use of **P**-matrices as substitute for **G** has been extensively used on macroevolutionary studies^30, 45, 53–61^. Direct comparisons of **P**-matrices among populations, species or higher taxa in a broad phylogenetic scale can be used to check for the constancy of **G**, and as evidence in support for the use of their phenotypic counterparts. Similarity among different species **P**-matrices constitutes strong evidence that the underlying **G** also remained stable^52^. This assumption was supported for phyllostomid bats by directly comparing the similarity of the phenotypic covariance and correlation patterns across species in a broad phylogenetic and taxonomically structured sample (*manuscript in preparation*). Given that the covariance matrices were considered similar among phyllostomid species, we used the **P**-matrices as substitute for **G** to investigate the evolutionary processes underlying their morphological diversification and in order to reconstruct the net selection gradients along their evolutionary history. These fulfilled premises allow us to extend the quantitative genetics theory into a macroevolutionary context.

### Testing hypothesis of evolution by genetic drift

According to Lande^28^ and Lofsvold^62^, the expected dispersal of average population phenotypes through multivariate genetic drift can be described by:

1 $${\bf{B}}={\bf{G}}(t/{N}_{e}),$$

where **B** is the between species variance-covariance matrix, **G** is the additive genetic variance-covariance matrix of the founding population from which the group of species originated, t is the elapsed time since divergence from the ancestral population in generations and N*e* is the effective size of the evolving populations. By this equation we can see that **G** is central for the evolution of complex morphological traits since its structure affects the future trajectory of phenotypes in response to genetic drift or selection (see Equations 4 and 5)^63, 64^. Given that we found a structural similarity among the **P**-matrices studied we used the within-group variance-covariance phenotypic matrix (**W**-matrix) as substitute for its genetic counterpart in equation 1, resulting in:

2 $${\bf{B}}{\rm{\alpha }}{\bf{W}}({\rm{t}}/{\rm{N}}e)$$

In this equation, N*e* and t are both constants for any particular comparison. Thus, the possibility of obtaining the observed pattern of phenotypic differentiation by genetic drift can be investigated by comparing **B** and **W** matrices^28, 29, 62, 65^. Below we describe two methods proposed by Ackerman and Cheverud^29^ to test hypothesis of evolution by genetic drift: the Regression test and the Correlation test. Both can be used as complementary approaches in testing hypotheses of neutral evolution because they allow for the investigation of different aspects of the covariance structure. While the regression test evaluates the proportionality between the within- and between-population variation, focusing on the diagonal of the **W**-matrix, the correlation test investigates co-selection (uncorrelated traits being selected together) focusing on the off-diagonal components of the **B**-matrix.

### The regression test

As previously described in Equations 1 and 2, if populations or species have diversified by random evolutionary processes, the expected phenotypic variation pattern between-groups would be proportional to the morphological variation pattern within them. Otherwise, the lack of proportionality between **B** and **W** may be an evidence of other evolutionary processes, such as directional selection^53, 66^. In order to simplify the comparisons between **B** and **W**, the **W**-matrices were reduced to their Principal Components (PCs), and ordered by their level of variance (corresponding to **W**-matrices PCs eigenvalues). Since these PCs are by definition uncorrelated with one another, the **W-**matrix represents a simple diagonal matrix with no covariances among elements. We calculated the PC scores for each species by multiplying their trait means by the standardized (norm equal to one) **W**-matrices eigenvectors. Then, the **B**-matrix was calculated as the variance among population mean projected on those PC scores. In order to take into account the correlation introduced due to phylogenetic non-independence, the **B**-matrix was estimated as the covariance of the Phylogenetic Independent Contrasts (PIC) of the original species data^31, 61, 67^.

Ackerman and Cheverud^29^ expressed the relationship between **B** and **W** (see Equation 1) as a log linear regression equation:

3 $$\mathrm{ln}({B}_{i})=\,\mathrm{ln}(t/{N}_{e})+{\rm{b}}\,\mathrm{ln}(W{\rm{i}}),$$

where B_i_ is the variance between groups in the PCs of **W** projected means (estimated here using phylogenetic independent contrasts), *W*i are the eigenvalues of **W**, b is the slope or the regression coefficient and t/N_e_ is the intercept of the regression line. If species diversification was produced by random evolutionary process, we expect b to be 1.0. On the other hand, a regression slope significantly different from 1.0 suggests that genetic drift is not sufficient to explain the observed diversification. In this case, slopes above 1.0 are an indication that one or more of the first PCs (frequently the first one) are more variable than expected by drift. This can happen through diversifying selection for the first PCs or by stabilizing selection on the later PCs. Slopes significantly below 1.0 occur when species are relatively highly divergent along minor PCs, and this can happen through strong diversifying selection along these PCs or by stabilizing selection on the remaining PCs. Finally, since the number of species presented in the analysis has an influence in the uncertainty of the confidence interval, we performed the regression test only to nodes encompassing more than five terminal taxa. This test is implemented in the R package “evolqg”^48^ (function TreeDriftTest)^29^.

### The correlation test

The idea behind this test is that in a phylogenetic context, an observed correlation among traits can arise by two distinct processes: genetic covariances or co-selection (two traits being selected together, regardless of the genetic correlation between them)^31, 68^. Because the within-group PCs are orthogonal to each other we expect the mean between-group PCs scores to also remain uncorrelated if genetic drift causes species diversification. However, under diversifying directional selection, the expected **B** matrix is:

4 $${\bf{B}}={\bf{GCG}},$$

where **G** is the genetic variance-covariance matrix (**W** matrix in the present study), **C** is the variance-covariance matrix among selection gradients for the traits^68, 69^. In the **C** matrix, the off-diagonal components represent the between traits covariance produced by selection. Equation 4 shows two potential sources of correlated evolution among traits: common inheritance (captured in **W**) and selective covariance (captured in **C**). Given that the within-group PCs are by definition uncorrelated with one another, the **W** matrix represents a simple diagonal matrix with no covariances among elements. Thus, any correlation in **B** must arise from **C**, suggesting co-selection of certain morphological traits within a clade.

For each node in the phylogeny, we projected the species mean on the PCs of **W** and calculated its scores. We then computed Pearson product-moment correlations between species scores on **W**-matrix eigenvectors using PIC^30, 31, 70^. Under a null hypothesis of random evolutionary process, we would expect no correlation between the species means in any two PCs being compared. We tested for significant correlations among the first several PCs scores for each comparison involving five or more taxa and rejected the null hypothesis of evolution through drift whenever significant correlations were found among at least one pair of PCs using Bonferroni criteria. We correlated *n* - 1 number of PCs, with *n* corresponding the number of taxa in the analysis. This test is implemented in the R package “evolqg”^48^ (function PCScoreCorrelation)^30^.

### Selection strength reconstruction

We estimated ancestral states for 35 cranial measurements along the phylogeny using the linear parsimony approach using Mesquite version 3.02^71^, which does not take into account the branch length when calculating ancestral values. We have also calculated ancestral states using a maximum-likelihood approach^72^ that assumes a Brownian motion model of evolution and includes the branch length in the estimates (function fastAnc of “phytools” R package^73^). Since both methods showed equivalent patterns (correlation estimated by both methods equals 0.86, *SI Appendix*, Fig. S7), we chose to present the results obtained by the linear parsimony ancestral reconstruction approach. After estimating the ancestral states, we calculated the response to selection vector ($\triangle z$) within each branch along the phylogeny as the difference vector between two subsequent nodes or between an extant species and its ancestor mean estimates. The net-selection gradient was then estimated based on Lande’s multivariate equation^28^:

5 $$\beta ={{\boldsymbol{W}}}^{-1}{\rm{\Delta }}{z}$$

where $\beta$ represents the selection gradient, $\triangle z$ is the vector of morphological change and ${{\boldsymbol{W}}}^{-1}$ is the inverse of the pooled within-group matrix for each node in the phylogeny.

Due to the fact that inverted matrices are dominated by small eigenvalues, causing bias in the estimation of selection gradients, we used an eigenvalue extention method for noise control on the **W**-matrices^74^. For this, we investigated the second derivative variance of the eigenvalues of each **W**-matrices. When the observed value stabilizes near zero, we replaced the eigenvalue of the subsequent eigenvectors to the stable one, using the function ExtendMatrix of the “evolqg” R package^48^. The magnitude of selection was then estimated as the norm of the mean standardized $\beta$-vector^75, 76^.

### Diversification of feeding specialization

Each of the subfamilies of Phyllostomidae is recognized mainly due to their dietary specializations and associated morphological adaptations^6, 40, 77^. Phyllostomidae are nested in a group of animalivorous bats, the superfamily Noctilionoidea^8^. Basal phyllostomids kept the animalivorous habits but with a significant difference, the gleaning habit (most other noctilionoids are aerial hawking bats). Phytophagous phyllostomids, both nectar feeding and frugivorous species, evolved in clades nested among ancestral animalivorous phyllostomids^32, 40, 78^. Previous studies of dietary diversification in phyllsotomid bats have described that the insectivorous ancestor of all phyllostomids also feed on plants^32, 40, 77^. Dietary information was obtained from the literature based on Nowak^79^, Ferrarezi and Gimenez^78^, Simmons^80^, Wetterer *et al*.^81^, Baker *et al*.^40^ and supplemented by personal experience (N. P. Giannini). The dietary information is therefore a product of our research of these sources for this paper. We organized the dietary information for the 57 species into eight categories: insectivory (includes insects and plant material in dietary habits), strict insectivory, hematophagy, omnivory, carnivory, nectarivory, frugivory and obligate frugivory. We mapped the different dietary regimes to the phylogenetic tree using the function “make.simmap” from “phytools” R package^73^, which simulates stochastic character histories, using Markov model based on the states assigned to the tips of the tree. We used “equal rate model” because it performed better in the reconstruction compared with the other models (AIC “equal rate model”: 116,07; AIC “symmetrical rates”: 147,62; AIC “all rates different”: 191,93).

## Electronic supplementary material

Supplementary Information
