# Supplementary material for: Intense natural selection preceded the invasion of new adaptive zones during the radiation of New World leaf-nosed bats
Source: Sci Rep. 2017 Sep 11;7:11076. doi: 10.1038/s41598-017-08989-6 (PMC5593990; doi:10.1038/s41598-017-08989-6)
Supplement: Supplementary file 1 — Supplementary Information [file 41598_2017_8989_MOESM1_ESM.pdf]

Supplementary Information Appendix

"Intense natural selection preceded the invasion of new adaptive zones during the radiation of New World leaf-nosed bats"

Daniela M. Rossoni[1*], Ana Paula A. Assis[2], Norberto P. Giannini[3] and Gabriel Marroig[1]

[1]*Department of Genetics and Evolutionary Biology, Biosciences Institute, University of São Paulo, Rua do Matão, 277, 05508-900, São Paulo, Brazil.*

[2]*Department of Ecology, Biosciences Institute, University of São Paulo, Rua do Matão, 277, 05508-900, São Paulo, Brazil.*

[3]*Unidad Ejecutora Lillo-CONICET, Miguel Lillo 251, Universidad Nacional de Tucumán, CP 4000, Argentina.*

*Corresponding author: Daniela M. Rossoni, daniela.rossoni@usp.br

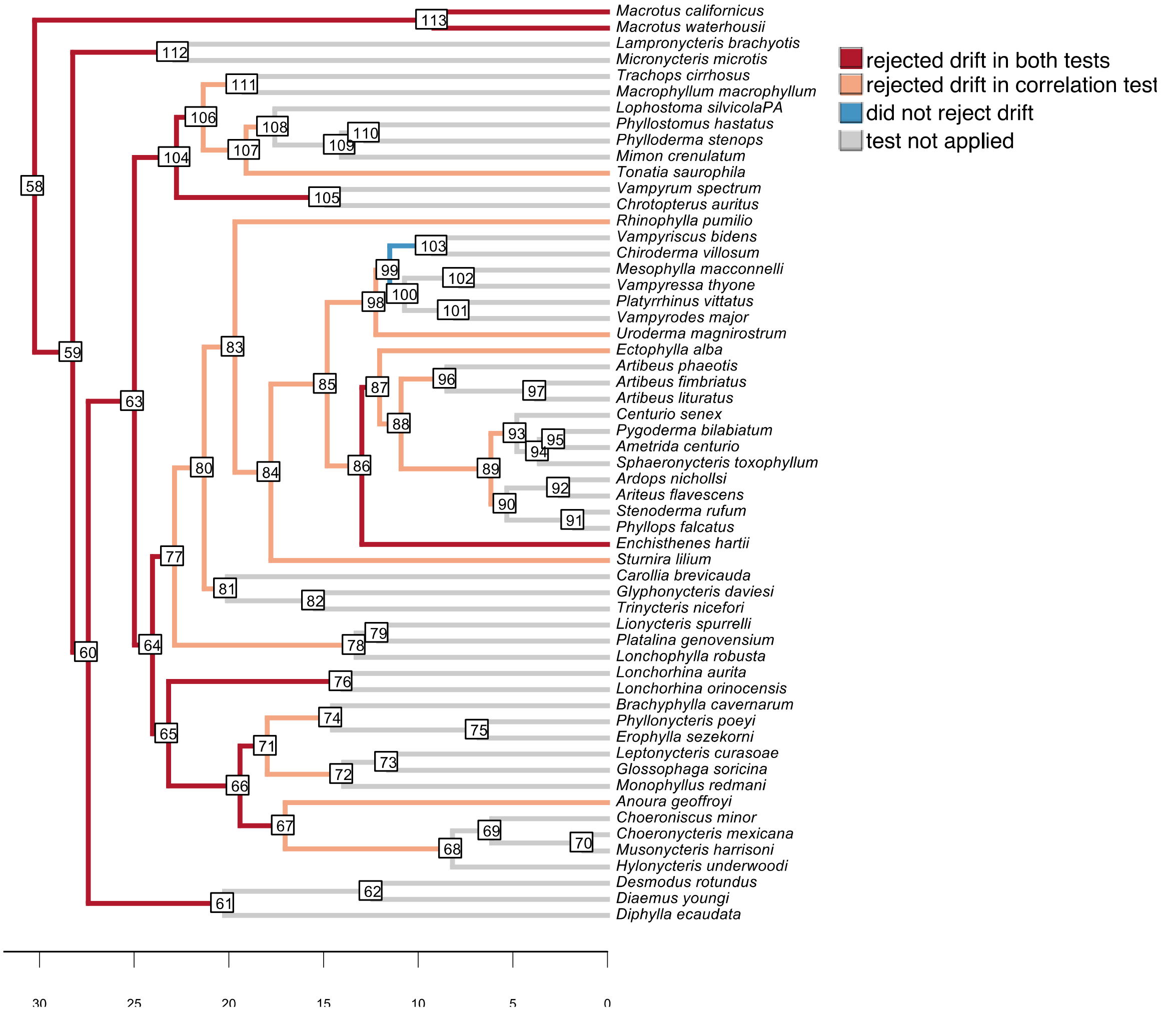

Figure S1. Results of genetic drift tests plotted on a phylogenetic tree for Phyllostomidae. Tests were not applied to nodes encompassing <5 terminal taxa (branches in gray). Phylogeny based on Rojas[1]. Scale bar: million years (Myr).

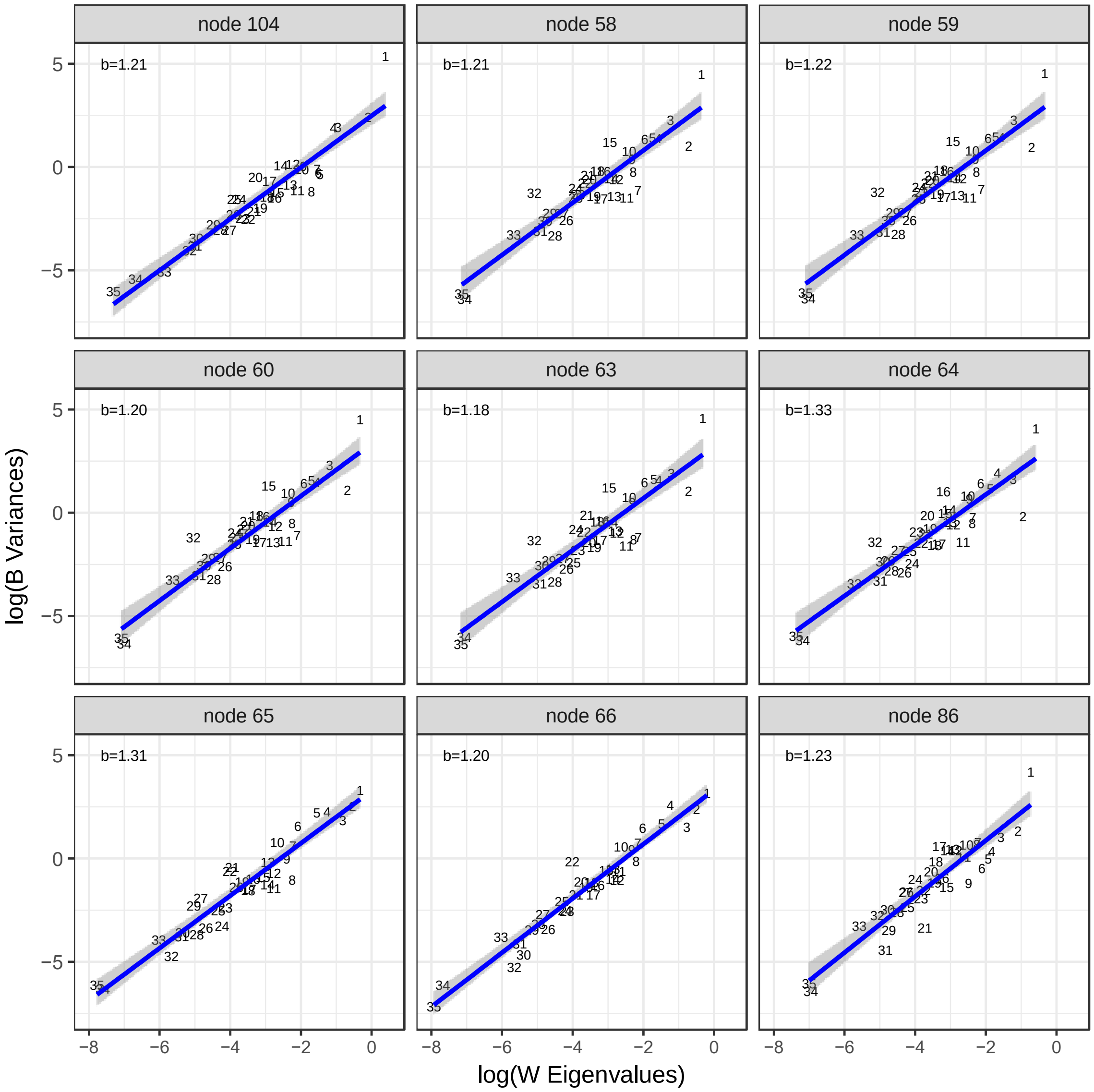

Figure S2. Regression test plots of between-groups variances (**B**) on within-group eigenvalues (**W**) and the associated 95% confidence intervals for nodes in the phylogeny where the genetic drift has been rejected. The blue line represents the estimated regression line with respective confidence interval. Regression slopes (b) are displayed in the left upper corner. Number labels inside the plots represent the principal components.

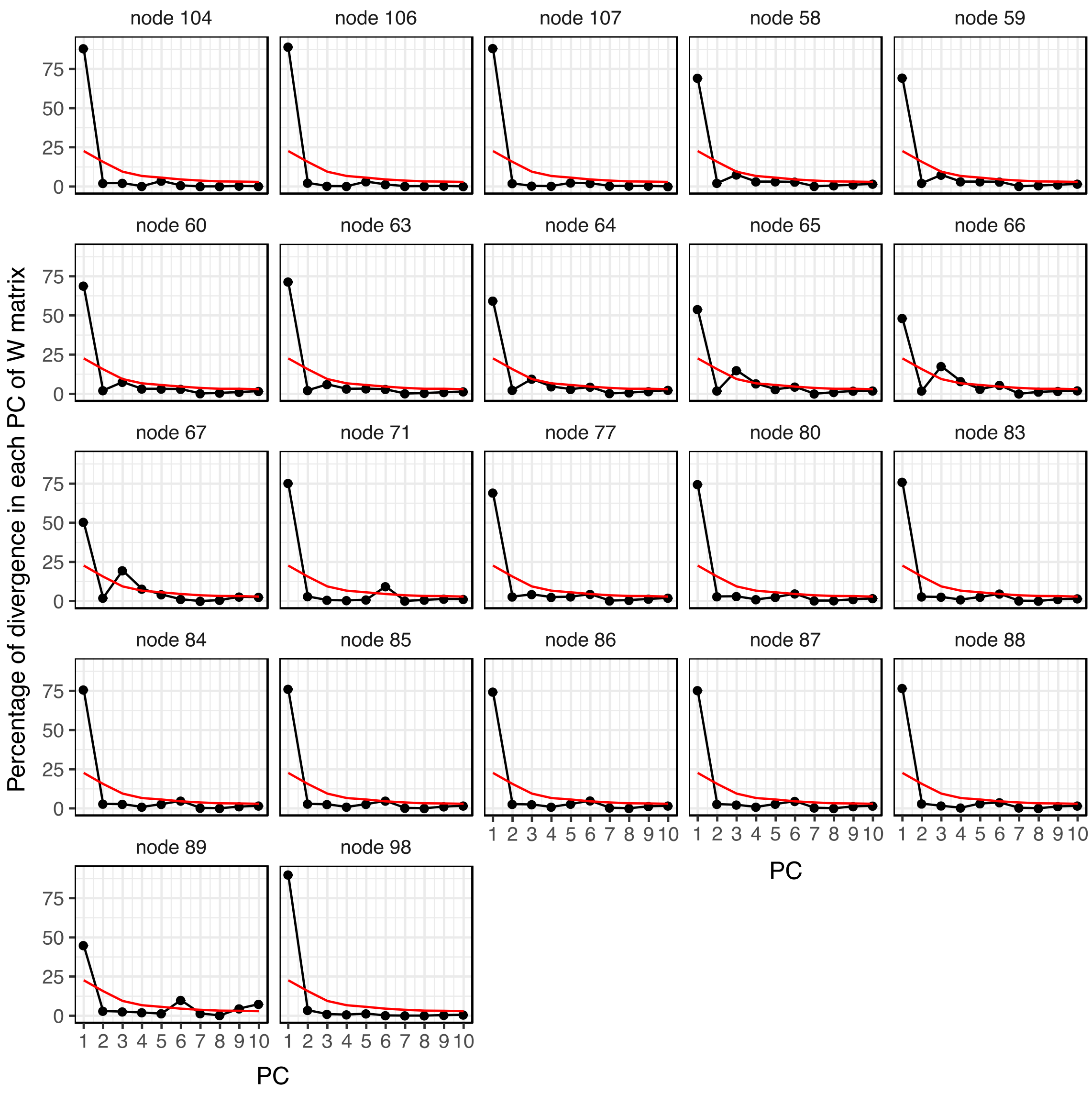

Figure S3. Directions of divergence between species (**B**) projected in the first 10 PCs of the ancestral **W**-matrix (black lines and dots). Red lines indicate the percentage of variance explained by each PC of the ancestral **W**-matrix (see Table S1). Note that most of the divergence observed between phyllostomid species is associated with the first principal component.

Table S1. Eigenvectors for the 10 PCs estimated from the ancestral **W**-matrix (node 58 in the Phyllostomidae phylogeny showed in Figure S1). The first column represents linear distances between the landmarks displayed in Figure S6. The regions and subregions, representing functional/developmental groups are also displayed. Boldface indicates the most extreme loading values.

Distances	Region	Subregion	PC1	PC2	PC3	PC4	PC5	PC6	PC7	PC8	PC9	PC10
ISPM	Oral	Face	-0.032	0.010	0.007	-0.004	-0.041	-0.016	-0.002	-0.036	-0.008	-0.025
ISNSL	Nasal	Face	-0.087	0.026	-0.004	0.056	-0.047	-0.050	0.053	**0.105**	0.069	-0.024
ISPNS	Oral, Nasal	Face	-0.275	0.083	**-0.114**	-0.059	**-0.348**	**-0.357**	-0.006	0.019	**0.206**	**-0.241**
PMZS	Oral	Face	-0.238	**0.113**	**-0.338**	0.063	0.062	0.012	0.087	**0.112**	**-0.211**	-0.088
PMZI	Oral	Face	-0.233	**0.101**	**-0.350**	0.027	**0.162**	-0.004	0.040	0.051	0.020	0.047
PMMT	Oral	Face	-0.194	0.052	**-0.128**	-0.038	**-0.102**	-0.023	0.043	0.085	-0.065	-0.083
NSLNA	Nasal	Face	-0.135	**-0.144**	**-0.219**	**-0.669**	**-0.186**	**0.230**	**-0.241**	**-0.182**	-0.040	-0.005
NSLZS	Nasal	Face	-0.242	**0.107**	**-0.299**	0.025	0.049	0.029	0.070	0.055	**-0.241**	-0.051
NSLZI	Oral, Nasal	Face	-0.258	0.088	**-0.306**	-0.003	**0.116**	0.022	0.021	0.040	-0.007	**0.118**
NABR	Cranial vault	Neurocranium	-0.037	**0.759**	**0.213**	**0.219**	-0.031	0.044	-0.040	0.014	-0.168	-0.038
NAPNS	Nasal	Face	-0.181	0.063	-0.025	0.077	**-0.203**	**-0.264**	-0.040	**0.148**	**0.361**	-0.030
BRPT	Cranial vault	Neurocranium	-0.054	**0.375**	**0.109**	**-0.136**	-0.095	**0.243**	**-0.404**	**-0.191**	0.086	**0.164**
BRAPET	Cranial vault	Neurocranium	-0.210	-0.034	0.079	**0.161**	**-0.136**	**0.299**	**-0.160**	0.036	**0.320**	**0.125**
PTAPET	Cranial vault	Neurocranium	-0.252	-0.046	**0.222**	-0.071	**0.198**	**0.147**	**0.118**	**0.120**	**0.311**	**-0.126**
PTBA	Cranial vault	Neurocranium	-0.297	-0.042	**0.214**	-0.075	**0.138**	-0.090	-0.009	-0.058	0.026	**0.170**
PTEAM	Cranial vault	Neurocranium	-0.233	-0.034	**0.240**	-0.093	**0.204**	**-0.129**	**0.129**	**-0.228**	-0.061	**-0.186**
PTZYGO	Zygomatic	Face	-0.216	-0.060	**0.179**	**-0.100**	**0.107**	**-0.112**	**0.194**	**0.183**	**-0.123**	**0.201**
PTTSP	Cranial vault, Zygomatic	Neurocranium, Face	-0.170	-0.028	**0.202**	**-0.104**	**0.223**	**-0.114**	**0.139**	0.036	0.089	0.059
ZSZI	Oral	Face	-0.057	-0.017	0.008	0.008	-0.083	0.023	-0.041	**0.114**	0.033	0.078
ZIMT	Oral	Face	-0.081	0.034	**-0.127**	0.032	**0.107**	0.027	0.032	-0.016	0.048	0.084

ZIZYGO	Zygomatic	Face	-0.061	-0.054	**0.292**	**-0.141**	**-0.417**	0.009	0.099	**0.327**	**-0.337**	-0.015
ZITSP	Zygomatic	Face	-0.075	-0.005	**0.170**	-0.079	**-0.336**	0.048	0.075	0.072	**-0.191**	**-0.209**
MTPNS	Oral	Face	-0.074	0.029	0.018	-0.021	**-0.180**	**-0.170**	-0.016	-0.057	**0.114**	**-0.134**
PNSAPET	Basicranium	Neurocranium	-0.097	0.005	0.038	-0.016	0.096	**0.553**	**0.164**	**0.196**	**-0.154**	**-0.183**
APETBA	Basicranium	Neurocranium	-0.055	0.003	-0.008	-0.001	-0.064	**-0.235**	**-0.131**	**-0.176**	**-0.282**	**0.305**
APETTS	Basicranium	Neurocranium	-0.040	0.010	0.043	-0.054	0.033	**0.197**	**0.105**	0.067	**0.160**	**-0.256**
BAEAM	Basicranium	Neurocranium	-0.126	0.003	0.008	-0.018	-0.088	0.054	-0.051	**0.196**	0.083	**0.320**
EAMZYGO	Zygomatic	Face	-0.054	0.040	-0.015	0.065	-0.004	0.019	0.000	**-0.454**	-0.005	**-0.482**
ZYGOTSP	Zygomatic	Face	-0.079	-0.011	-0.009	0.010	-0.097	0.013	0.043	0.028	**-0.125**	-0.012
LDAS	Cranial vault	Neurocranium	-0.068	0.050	-0.003	0.095	**-0.247**	**0.199**	**0.417**	**-0.303**	**0.188**	**0.237**
BRLD	Cranial vault	Neurocranium	-0.275	**-0.426**	0.017	**0.591**	**-0.180**	**0.159**	**-0.231**	**-0.150**	**-0.178**	-0.005
OPILD	Cranial vault	Neurocranium	-0.086	0.050	0.021	-0.044	**-0.180**	0.076	**0.414**	**-0.385**	-0.010	**0.277**
PTAS	Cranial vault	Neurocranium	-0.322	-0.038	**0.280**	-0.057	**0.240**	-0.082	**-0.269**	**-0.166**	**-0.172**	-0.021
JPAS	Basicranium	Neurocranium	-0.065	0.004	0.010	0.011	0.004	0.012	**-0.248**	0.055	-0.021	0.045
BAOPI	Basicranium	Neurocranium	-0.045	0.015	0.022	0.035	-0.010	0.094	**-0.180**	**0.148**	**0.189**	-0.032
**Eigenvalues**			0.70	0.48	0.29	0.20	0.17	0.14	0.11	0.10	0.09	0.09
**% variance**			29.16	20.00	12.00	8.33	7.08	5.83	5.00	4.16	4.08	3.75

Table S2. Projection of species means on the first principal component of ancestral **W**-matrix (node 58 in the Phyllostomidae phylogeny showed in Figure S1) and geometric means per species. The observed Pearson correlation between PC1 and geometric mean is 0.97, indicating that PC1 is an allometric size component.

Species	Geometric_mean	PC1 W58
Ametrida_centurio	3.64	24.53
Anoura_geoffroyi	5.05	40.15
Ardops_nichollsi	5.12	35.11
Ariteus_flavescens	4.44	30.14
Artibeus_fimbriatus	7.07	51.76
Artibeus_lituratus	7.13	51.46
Artibeus_phaeotis	4.53	32.12
Brachyphylla_cavernarum	6.57	48.23
Carollia_brevicauda	4.85	34.90
Centurio_senex	4.83	32.15
Chiroderma_villosum	5.73	41.20
Choeroniscus_minor	4.21	36.07
Choeronycteris_mexicana	5.27	44.98
Chrotopterus_auritus	7.60	55.45
Desmodus_rotundus	5.41	38.37
Diaemus_youngi	5.40	36.77
Diphylla_ecaudata	4.97	33.99
Ectophylla_alba	3.80	27.04
Enchisthenes_hartii	4.63	33.42
Erophylla_sezekorni	4.66	35.39
Glossophaga_soricina	4.11	31.60
Glyphonycteris_daviesi	5.66	41.44
Hylonycteris_underwoodi	4.10	33.05
Lampronycteris_brachyotis	4.54	33.09
Leptonycteris_curasoae	5.29	41.56
Lionycteris_spurrelli	3.88	29.72
Lonchophylla_robusta	5.17	40.97
Lonchorhina_aurita	4.29	30.72
Lonchorhina_orinocensis	3.82	27.84
Lophostoma_silvicolum	5.59	40.15
Macrophyllum_macrophyllum	3.60	25.75
Macrotus_californicus	4.48	33.82
Macrotus_waterhousii	4.46	33.42
Mesophylla_macconnelli	3.90	28.00
Micronycteris_microtis	3.73	27.29
Gardnerycteris_crenulatum	4.50	32.39
Monophyllus_redmani	4.51	34.70
Musonycteris_harrisoni	5.37	48.27
Phylloderma_stenops	6.54	46.50

Phyllonycteris_poeyi	4.74	35.68
Phyllops_falcatus	4.60	31.97
Phyllostomus_hastatus	8.08	58.48
Platalina_genovensium	5.65	45.99
Platyrrhinus_vittatus	7.17	52.52
Pygoderma_bilabiatum	4.74	32.78
Rhinophylla_pumilio	3.95	28.51
Sphaeronycteris_toxophyllum	4.04	27.44
Stenoderma_rufum	5.06	34.13
Sturnira_lilium	5.05	36.46
Tonatia_saurophila	5.85	41.79
Trachops_cirrhosus	6.04	44.37
Trinycteris_nicefori	4.12	30.33
Uroderma_magnirostrum	5.24	38.94
Vampyriscus_bidens	4.39	31.51
Vampyressa_thyone	4.11	29.27
Vampyrodes_major	6.46	46.16
Vampyrum_spectrum	10.10	75.37

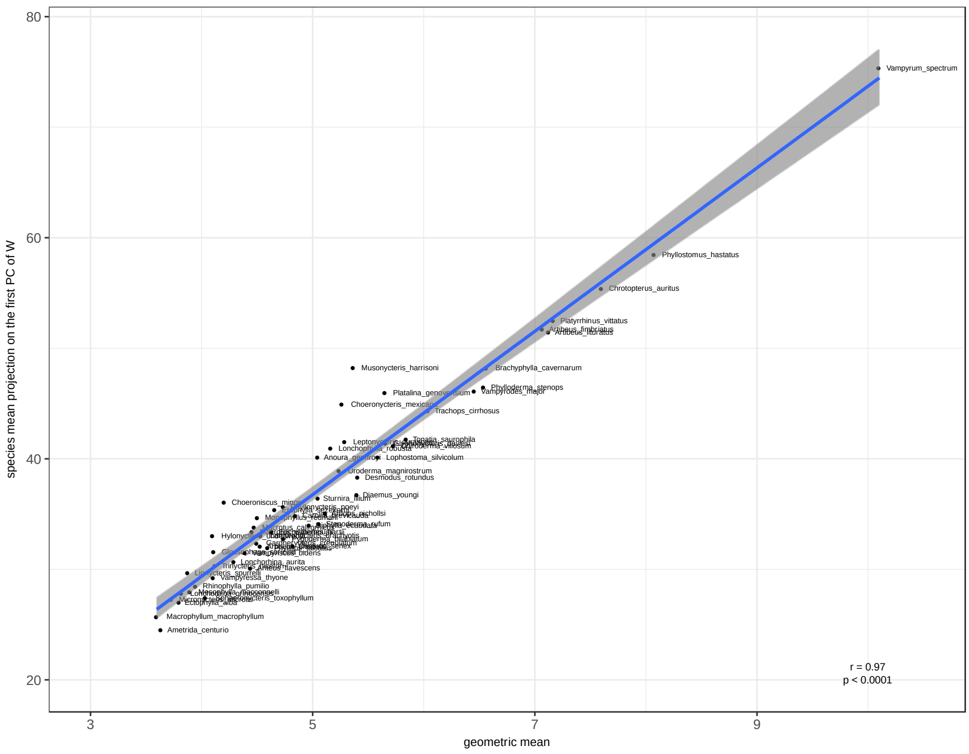

Figure S4. Correlation between projection of species means on the first principal component of ancestral **W**-matrix (node 58 in the Phyllostomidae phylogeny showed in Figure S1) and geometric means.

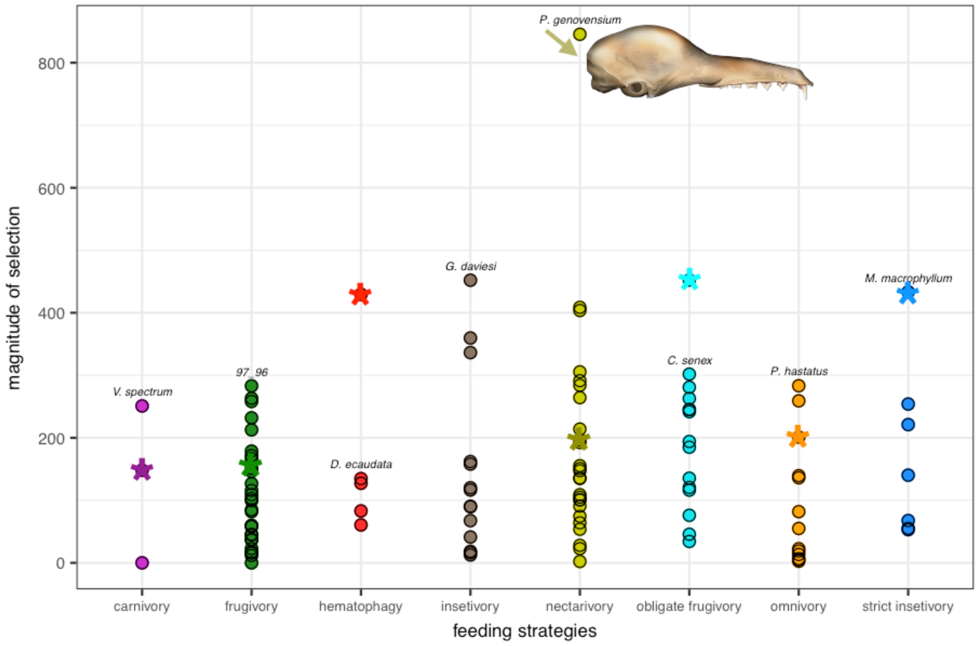

Figure S5. Distribution of the magnitude of selection, estimated as the norm of the selection gradient vector, in eight dietary categories. Circles indicate branches in the phylogeny, and the symbol (*) indicates dietary transitions. Species names correspond to greatest magnitudes of selection for each feeding strategies (*Vampyrum spectrum*: carnivory; *Ectophylla alba*: frugivory; *Diphylla ecaudata*: hematophagy; *Glyphonycteris daviesi*: insetivory; *Platalina genovensium*: nectarivory; *Centurio senex*: obligate frugivory; *Phyllostomus hastatus*: omnivory; *Macrophyllum macrophyllum*: strict insetivory). Skull: *Platalina genovensium*. Dietary information was obtained from the literature based on Nowak[2], Ferrarezi and Gimenez[3], Simmons[4], Wetterer et al.[5], Baker et al.[6], and supplemented by personal experience (N. P. Giannini).

Table S3. 57 species of phyllostomid bats measured in this study, with respective sample sizes (N). Sources of variation controlled during the pooled within-species variance-covariance matrix estimation are presented. S: sex, L: locality.

Species	Model	N	Species	Model	N
Micronycteris microtis	-	60	*Ariteus flavescens*	S	59
Lampronycteris brachyotis	-	14	*Phyllops falcatus*	S + L	50
Lonchorhina aurita	-	39	*Stenoderma rufum*	S	60
Lonchorhina orinocensis	S	40	*Enchisthenes hartii*	S + L	62
Lonchophylla robusta	S	68	*Chiroderma villosum*	S	60
Lionycteris spurrelli	-	60	*Vampyriscus bidens*	-	48
Platalina genovensium	-	6	*Uroderma magnirostrum*	-	60
Monophyllus redmani	S	58	*Vampyressa thyone*	S + L	62
Leptonycteris curasoae	S	61	*Mesophylla macconnelli*	S + L	60
Glossophaga soricina	-	35	*Vampyrodes major*	-	62
Brachyphylla cavernarum	S + L	52	*Platyrrhinus vittatus*	-	59
Erophylla sezekorni	S	60	*Glyphonycteris daviesi*	-	4
Phyllonycteris poeyi	S	60	*Trinycteris nicefori*	-	61
Anoura geoffroyi	-	39	*Carollia brevicauda*	S	69
Hylonycteris underwoodi	-	31	*Macrophyllum macrophyllum*	S + L	51
Choeronycteris mexicana	-	51	*Trachops cirrhosus*	-	60
Musonycteris harrisoni	-	5	*Tonatia saurophila*	S + L	50
Choeroniscus minor	-	33	*Lophostoma silvicola*	S + L	70
Rhinophylla pumilio	S + L	52	*Phyllostomus hastatus*	S + L	64
Sturnira lilium		33	*Phylloderma stenops*	S + L	34
Artibeus lituratus	S	40	*Mimon crenulatum*	-	39
Artibeus fimbriatus	-	51	*Chrotopterus auritus*	S + L	58
Artibeus phaeotis	-	45	*Vampyrum spectrum*	S + L	41
Ectophylla alba	-	11	*Diphylla ecaudata*	S + L	56
Centurio senex	S + L	66	*Diaemus youngi*	S + L	59
Sphaeronycteris toxophyllum	S	64	*Desmodus rotundus*	S	86
Ametrida centurio	S + L	48	*Macrotus californicus*	S	59
Pygoderma bilabiatum	S	57	*Macrotus waterhousii*	S	60
Ardops nichollsi	-	6			

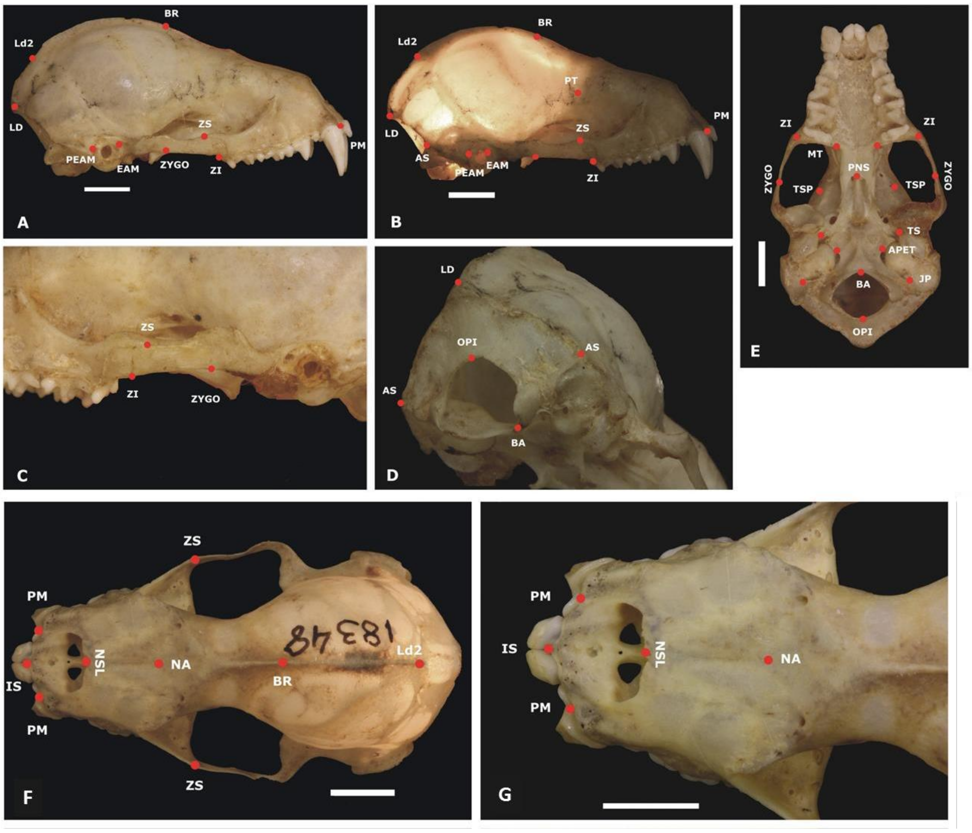

Figure S6. Cranial anatomical landmarks in *Chrotopterus auritus* (A-E; MZUSP 3077) and *Phyllostomus hastatus* (F-G; MZUSP 18348). A and B - lateral view. C - oblique dorsolateral view with jugal bone in detail. D - oblique occipital view. E - ventral view. F - dorsal view. G - dorsal view with nasal and frontal bones in detail. Scale bar = 5 mm.

Table S4. Anatomical landmarks recorded from skulls by using a 3D digitizer with descriptions and anatomical references. The anatomical references (bones and sutures involved) are based on Evans and Evans[7] and Giannini et al[8]. Names of the skull bones are given in English and the sutures are given in Latin terminology based on the fourth edition of the Nomina Anatomica Veterinaria[9].

Landmarks	Descriptions	Bones involved	Sutures involved
**IS**	Intradentale superior	premaxilla	*sutura interincisiva*
**PM**	Premaxillari-maxillari suture at the alveolus	premaxila; maxilla	*sutura maxilloincisiva*
**NSL**	Nasale	nasal	*sutura internasalis*
**NA**	Nasion	nasal; frontal	*sutura frontonasalis*
**BR**	Bregma	frontal; parietal	*sutura sagittalis*; *sutura coronalis*
**PT**	Pterion	frontal; parietal; orbitosphenoid	*sutura sphenofrontalis; sutura sphenoparietalis*
**ZS**	Zygomaxillare superior	maxilla; jugal	*sutura zygomaticomaxillaris*
**ZI**	Zygomaxillare inferior	maxilla; jugal	*sutura zygomaticomaxillaris*
**MT**	Maxillary tuberosity	palatine; maxilla	*sutura palatomaxillaris*
**PNS**	Posterior nasal spine	palatine	*sutura interpalatina*
**APET**	Anterior petrous temporal	basisphenoid; basioccipital	*spheno-occipital synchondrosis*
**BA**	Basion	basioccipital	-
**OPI**	Opisthion	supraoccipital	-
**EAM**	External auditory meatus	maleus	-
**PEAM**	Posterior auditory meatus	maleus	-
**ZYGO**	Inferior zygo-temporal suture	jugal; squamosal	*sutura temporozygomatica*
**TSP**	Temporo-spheno-parietal junction	parietal; alisphenoid; squamosal	*sutura squamosa; sutura sphenoparietalis*
**TS**	Temporo-sphenoidal junction at petrous	squamosal; alisphenoid	*sutura sphenosquamosa*
**JP**	Jugular foramen	basioccipital; exoccipital	*posterior basicochlear comissure*
**LD**	Lambda	interparietal; supraoccipital	*sutura occipitointerparietalis (lambdoidea)*
**AS**	Asterion	parietal; supraoccipital	*sutura occipitoparietalis*

Table S5. 35 linear distances and its classification on functional/developmental group and skull regions.

Distances	Functional/Developmental group	Region
IS-PM	Oral	Face
IS-NSL	Nasal	Face
IS-PNS	Oral, Nasal	Face
PM-ZS	Oral	Face
PMZI	Oral	Face
PM-MT	Oral	Face
NSL-NA	Nasal	Face
NSL-ZS	Nasal	Face
NSL-ZI	Oral, Nasal	Face
NA-BR	Cranial vault	Neurocranium
NA-PNS	Nasal	Face
BR-PT	Cranial vault	Neurocranium
BR-APET	Cranial vault	Neurocranium
PT-APET	Cranial vault	Neurocranium
PT-BA	Cranial vault	Neurocranium
PT-EAM	Cranial vault	Neurocranium
PT-ZYGO	Zygomatic	Face
PT-TSP	Cranial vault, Zygomatic	Neurocranium, Face
ZS-ZI	Oral	Face
ZI-MT	Oral	Face
ZI-ZYGO	Zygomatic	Face
ZI-TSP	Zygomatic	Face
MT-PNS	Oral	Face
PNS-APET	Basicranium	Neurocranium
APET-BA	Basicranium	Neurocranium
APET-TS	Basicranium	Neurocranium
BA-EAM	Basicranium	Neurocranium
EAM-ZYGO	Zygomatic	Face
ZYGO-TSP	Zygomatic	Face
LD-AS	Cranial vault	Neurocranium
BR-LD	Cranial vault	Neurocranium
OPI-LD	Cranial vault	Neurocranium
PT-AS	Cranial vault	Neurocranium
JP-AS	Basicranium	Neurocranium
BA-OPI	Basicranium	Neurocranium

Table S6. Symbols used throughout the text and their descriptions.

Symbols	Descriptions
**P**-matrices	phenotypic variance-covariance matrices; or the covariance matrices for a terminal taxa in a phylogenetic tree.
**W**-matrices	ancestral within-group covariance matrices for each node of a phylogenetic tree as weighted averages of species P-matrices.
ancestor **W**-matrix	within-group covariance matrix at the root of a phylogenetic tree used as an approximation of the ancestral G.
Δz	vector of differences in the averages of any two groups; or the vector of response to selection.
**B**	between groups variance-covariance matrix estimated on phylogenetic independent contrasts.
**G**	additive genetic variance-covariance matrix.
β	selection gradient.
t	elapsed time since divergence from the ancestral population in generations.
N*e*	effective size of the evolving populations.
W	eigenvalues of within-group covariance matrices (**W**).
b	slope of the regression coefficient.
**C**	variance-covariance matrix among selection gradients for the traits.

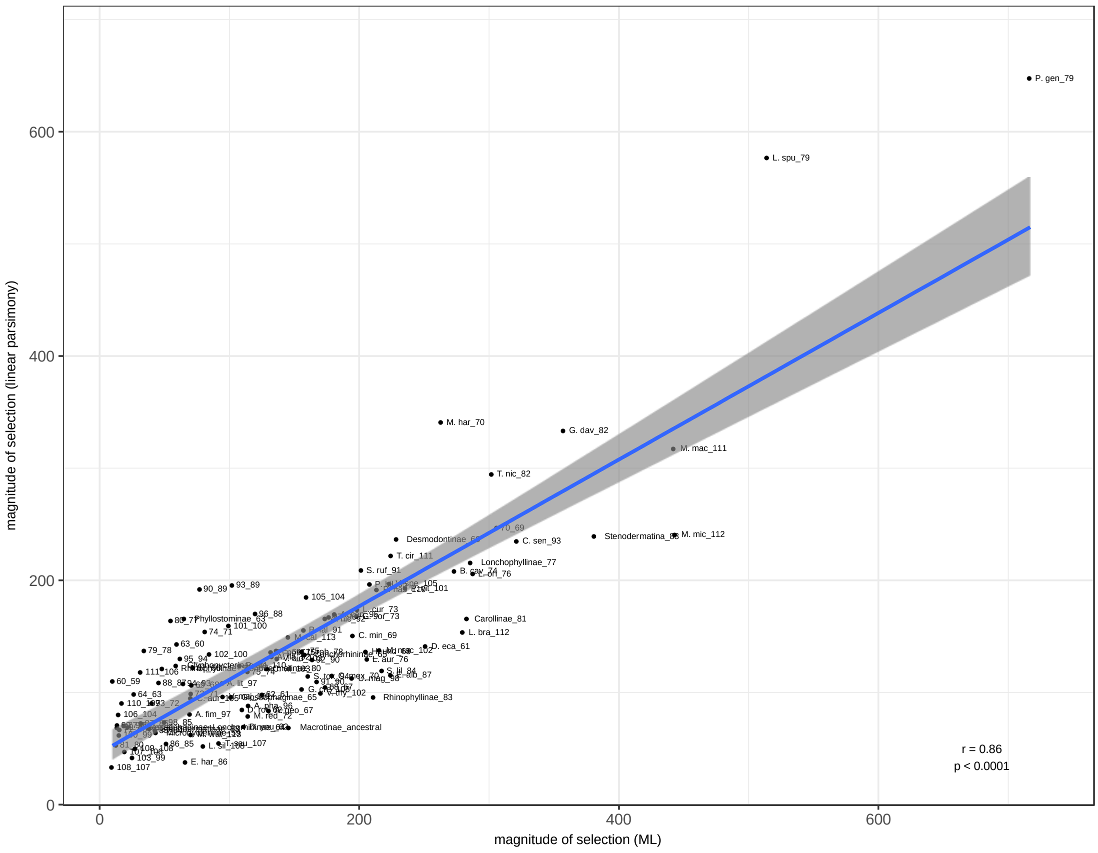

Figure S7. Correlation between selection strength values reconstructed based on linear parsimony approach using Mesquite version 3.02[10] and using a maximum likelihood approach[11] (function fastAnc of "phytools" R package[12]).

References

1. Rojas, D., Warsi, O. M. & Dávalos, L. M. Bats (Chiroptera: Noctilionoidea) Challenge a Recent Origin of Extant Neotropical Diversity. *Syst. Biol.* **65,** 432–448 (2016).
2. Nowak, R. M. & Walker, E. P. *Walker's bats of the world*. (Johns Hopkins University Press, 1994).
3. Ferrarezi, H. & Gimenez, E. . Systematic patterns and the evolution of feeding habits in Chiroptera (Archonta: Mammalia). *J. Comp. Biol.* **1,** 75–94 (1996).
4. Simmons, N. B. Order Chiroptera. in *Mammal Species of the World: a taxonomic and geographic reference* **1,** 312–529 (2005).

5. Wetterer, A. ., Rockman, M. V. & Simmons, N. B. Phylogeny of phyllostomid bats (Mammalia:Chiroptera): data from diverse morphological systems, sex chromosomes, and restriction sites. *Bull. Am. Mus. Nat. Hist.* **248,**
6. Baker, R. J., Bininda-Emonds, O. R. P., Mantilla-Meluk, H., Porter, C. A. & Van Den Bussche, R. A. Molecular time scale of diversification of feeding strategy and morphology in New World Leaf-Nosed Bats (Phyllostomidae): a phylogenetic perspective. in *Evolutionary History of Bats* (eds. Gunnell, G. F. & Simmons, N. B.) 385–409 (Cambridge University Press, 2012).
7. Evans, H. E. & Miller, M. E. *Miller's anatomy of the dog*. (W.B. Saunders, 1993).
8. Giannini, N. P., Wible, J. R. & Simmons, N. B. On the Cranial Osteology of Chiroptera. I. Pteropus (Megachiroptera: Pteropodidae). *Bull. Am. Mus. Nat. Hist.* **295,** 1–134 (2006).
9. *Nomina anatomica veterinaria*. (The Committees ; Distributed by Dept. of Veterinary Anatomy, Cornell University, 1994).
10. Maddison, W. P. & Maddison, D. R. *Mesquite: A modular system for evolutionary analysis Available at: http://mesquiteproject.org.* (2015).
11. Schluter, D., Price, T., Mooers, A. O. & Ludwig, D. Likelihood of ancestor states in adaptive radiation. *Evolution* **51,** 1699–1711 (1997).
12. Revell, L. J. phytools: an R package for phylogenetic comparative biology (and other things): phytools: R package. *Methods Ecol. Evol.* **3,** 217–223 (2012).
